# Sedentary Behavior in Preschoolers: How Many Days of Accelerometer Monitoring Is Needed?

**DOI:** 10.3390/ijerph121013148

**Published:** 2015-10-20

**Authors:** Wonwoo Byun, Michael W. Beets, Russell R. Pate

**Affiliations:** 1 Department of Health, Nutrition, and Exercise Sciences, Department of Public Health, North Dakota State University, Fargo, ND 58108, USA; 2 Department of Exercise Science, Arnold School of Public Health, University of South Carolina, Columbia, SC 29208, USA; E-Mails: beets@mailbox.sc.edu (M.W.B.); rpate@mailbox.sc.edu (R.R.P.)

**Keywords:** reliability, activity monitor, children, preschool, sedentary

## Abstract

The reliability of accelerometry for measuring sedentary behavior in preschoolers has not been determined, thus we determined how many days of accelerometry monitoring are necessary to reliably estimate daily time spent in sedentary behavior in preschoolers. In total, 191 and 150 preschoolers (three to five years) wore ActiGraph accelerometers (15-s epoch) during the in-school (≥4 days) and the total-day (≥6 days) period respectively. Accelerometry data were summarized as time spent in sedentary behavior (min/h) using three different cutpoints developed for preschool-age children (<37.5, <200, and <373 counts/15 s). The intraclass correlations (ICCs) and Spearman-Brown prophecy formula were used to estimate the reliability of accelerometer for measuring sedentary behavior. Across different cutpoints, the ICCs ranged from 0.81 to 0.92 for in-school sedentary behavior, and from 0.75 to 0.81 for total-day sedentary behavior, respectively. To achieve an ICC of ≥0.8, two to four days or six to nine days of monitoring were needed for in-school sedentary behavior and total-day sedentary behavior, respectively. These findings provide important guidance for future research on sedentary behavior in preschool children using accelerometry. Understanding the reliability of accelerometry will facilitate the conduct of research designed to inform policies and practices aimed at reducing sedentary behavior in preschool children.

## 1. Introduction

Childhood obesity is a significant public health concern because of its long-term detrimental health effects [1,2,3,4]. The rapid increase of obesity rates in American children and youth are known to be very difficult to reverse [5]. According to recent data from a representative sample, a third of American children and youth are classified as either overweight or obese [6]. In addition, childhood obesity is associated with numerous adverse health outcomes including type 2 diabetes, insulin resistance, hypertension, dyslipidemia, fatty liver disease, obstructive sleep apnea, and psychosocial difficulties in adulthood [7,8]. Given that obesity tracks into adulthood [9,10], efforts to prevent childhood obesity cannot be overemphasized.

Contemporary children spend a significant amount of their waking hours in sedentary behavior and this may result in negative health consequences. Recent studies reported that the majority of North American children engage in sedentary behavior more than the recommended levels [11,12,13,14,15,16], which are ≤2 h per day of TV watching [17] and <45 min per hour of sedentary behavior (equal to ≥15 min per hour of total physical activity) in childcare centers [18]. Accumulating evidence has recently shown that excessive sedentary behavior in childhood is a major contributor for obesity [19,20,21,22,23,24] and poor metabolic health [25,26,27,28,29], and it also impacts obesity and metabolic health in adulthood [30,31]. Therefore, the improvement of assessment methods for monitoring and reducing sedentary behavior is an important public health priority.

Accelerometry has been considered the method of choice for objective measurement of physical activity and sedentary behavior in children [32,33]. Its popularity is primarily due to its unobtrusiveness, light weight, small size, and ability to measure intensity, duration, and frequency of activities that children engaged in. In addition, accelerometery-derived activity data are less likely to be biased by recall or researcher bias. A recent study determined the reliability of measures of sedentary behavior in six- to eight-year-old elementary school children, and found that five days of accelerometry monitoring was necessary to reliably measure sedentary behavior [34]. However, the application of accelerometry in preschool-age (three- to five-year-old) children is relatively new, and little research has examined the reliability of accelerometry for measuring sedentary behavior among this population.

Using an Intraclass Correlation Coefficient (ICC) of ≥0.8 as the standard for acceptable reliability, previous studies determined the reliability of accelerometery-derived physical activity (PA) in children (aged 3–17 years), and reported that the recommended number of days of accelerometry monitoring to reliably measure moderate-to-vigorous PA varies from 4 to 10 days [35,36,37,38]. To our knowledge, however, no study has assessed the reliability of accelerometry-derived sedentary behavior in three- to five-year-old preschool children.

In addition to reliability, an important methodological issue in measuring sedentary behavior via accelerometry is the choice of cutpoints. Several cutpoints for preschool-age children have been used in previous studies, including <26 counts/15 s [39], <37.5 counts/15 s [40], <200 counts/15 s [40], <275 counts/15 s [32], <302 counts/15 s (for 4 years), [41] and <373 counts/15 s [42]. Depending on the cutpoint, the estimated time spent in accelerometry-derived sedentary behavior varies markedly (e.g., 343.2 min/day to 617.6 min/day) [42]. This suggests that the reliability of accelerometry-derived sedentary behavior differs when different cutpoints are used. Therefore, the purpose of this study was to determine how many days of accelerometry monitoring are necessary to reliably estimate daily time spent in sedentary behavior in preschool children when applying three different accelerometry cutpoints.

## 2. Experimental Section

### 2.1. Study Design

A cross-sectional study design was employed, and analyses were performed using data from the Children’s Activity and Movement in Preschool Study (CHAMPS). Details regarding the design of CHAMPS are available elsewhere [43]. Briefly, activity data were collected over 8–10 consecutive days in a sample of preschool children. Trained data collectors recorded preschool arrival and departure times for each child, so that each child’s daily activity data can be summarized as in-school sedentary behavior monitored during school hours, and total-day sedentary behavior monitored during and after school hours. For the analyses in the current study, we identified the subsets of the overall sample (*N* = 331) according to the activity monitored (in-school and total day) as follows: children who had at least 4 valid days (weekdays) of in-school sedentary behavior data (*N* = 191), or at least 6 valid days (including weekend days) of total-day sedentary behavior data (*N* = 150), respectively. A total of 139 children were identified in both subsamples (overlap sample).

### 2.2. Participants

The participants in this study were 3- to 5-year-olds preschool children. Using a stratified random sampling procedure, a total of 22 preschools (11 commercial, 7 religious, and 4 Head Start) agreed to participate in CHAMPS. The 22 preschools were recruited from the greater Columbia, South Carolina, USA, an area that includes a wide range of ethnic and socioeconomic backgrounds. The number of participants per preschool ranged from 14 to 33 children. Written informed consent was obtained from children’s parents or guardians prior to collection of data. The study was approved by the Institutional Review Board at the University of South Carolina.

### 2.3. Sedentary Behavior

Sedentary behavior was measured using ActiGraph accelerometers (ActiGraph model 7164, Shalimar, FL, USA). Accelerometers were initialized to save data in 15-s intervals (epochs) in order to more effectively capture the spontaneous movement of 3- to 5-year-old children. Parents were instructed to help their child to wear the accelerometers on an elastic belt on the right hip (anterior to the iliac crest). Parents received information about the monitor and instructions for helping their child wear the monitor over a two-week period. Accelerometry data were summarized in terms of activity counts per 15 s (cts/15 s).

To determine whether the number of days necessary to reliably estimate accelerometry-derived sedentary behavior in preschool children differs by application of selected sets of cutpoints, the count data were reduced using three different activity intensity cutpoints developed specifically for 3- to 5-year old children to categorize each interval as sedentary. These three cutpoints are <37.5 cts/15 s [40], <200 cts/15 s [40], and <373 cts/15 s [42]. Cumulative time (min/h) spent in sedentary behavior was then calculated using each child’s wear time as the divisor. Sixty-minutes of consecutive zeros were considered as non-wear time [13,44,45,46]. For in-school activity data, children must have attended school for at least 5 h on that day. Occasional (≈5%) missing entry and exit times were imputed based on the child’s other data (usual times entered on the consent form, entry and exit times on other days, and school average entry and exit times). Days that children were absent from preschool and on which total wear time was <6 h or ≥18 h (*i.e.*, monitor malfunction) were excluded from the analysis because those do not represent typical days.

### 2.4. Demographic and Anthropometric Characteristics

Children’s age, gender, race/ethnicity, and socioeconomic status were reported by a parent or guardian using a parent survey. Weight was measured to the nearest 0.1 kg using an electronic scale, and height was measured to the nearest 1 mm using a stadiometer, after the child had removed shoes and outer clothing. Parent education (≤High school, >High school) was measured as a surrogate indicator of socioeconomic status. Body Mass Index (BMI) was calculated (kg/m^2^) from the averages of both height and weight.

### 2.5. Statistical Analysis

Descriptive statistics (Mean, SD, and percent) were calculated for the subsets of the overall sample according to the activity monitored (in-school and total day). The coefficient of variance (CoV = (SD/mean) × 100) was calculated to describe between-individual variability [47]. A repeated measures analysis of variance (ANOVA) was used to determine if the amount of time spent in sedentary behavior is different across days of the week. Specifically, the difference in sedentary behavior was estimated by the PROC MIXED procedure in SAS, including gender, preschools, and gender × days of the week as the covariates, and a compound symmetric as the covariance structure. If an overall *F*-test was significant, multiple pairwise comparisons with Tukey adjustment were performed to determine differences in sedentary behavior between days. The LS MEANS statement with the PDIFF option was also included for pairwise comparisons in the PROC MIXED procedure.

The ICC was used to determine the reliability of the accelerometry-derived sedentary behavior and calculated using variance estimates from the two-way repeated ANOVA model and the formula below [48]:

*R* = (*MS_s_ − MS_i_)/[MS_s_ + (K/K’ − 1)(MS_i_*)]

where *MS_s_* is the mean square subject indicating the between-subject variability, *MS_i_* is the mean square interaction indicating variability interaction between the between-subject and within-subject variability, *K* is the number of days administered, *K’* is the number of days for which R is estimated, and *R* is the ICC.

The number of days of accelerometry monitoring required to reliably estimate sedentary behavior in preschool children was estimated using the Spearman-Brown prophecy formula below [48]:

r^*^_xx’_ = *K(r_xx’_)/[1 + (K − 1)(r_xx’_)]*

where *r_xx’_ is* the estimated reliability (ICC) of the test, r^*^_xx’_ is the desired reliability (ICC) of the lengthened test, and *K* is the number of times (days) the length of the test has been increased. More specifically, the ICC of 0.8 was considered as the desired reliability cutoff to determine the minimum number of days necessary to reliably estimate sedentary behavior [49].

The Standard Error of Measurement (SEM) was calculated to examine the precision of measured sedentary behavior from the estimated ICCs. The following formula was used [50]:

$$SEM=S_{x}\;\sqrt{\left(1-r_{xx’}\right)}$$

where *S_x_* is standard deviation of observed score, and *r_xx’_* is the estimated reliability (ICC) of the test. For all statistical analyses, the SAS statistical program, version 9.2 (SAS Institute, Cary, NC, USA) was used and the alpha level of 0.05 was considered as statistical significance.

## 3. Results

### 3.1. General Characteristics

Demographic characteristics of the participants and the average time the children wore the accelerometers are shown in Table 1. Approximately half of the children in this study were girls and half were African American. On average, the children wore the accelerometers for a total of 12.1 h/day and for 8.4 h/day while in school. The distribution of age, gender, race, and BMI was not different between two subsets of the overall sample (in-school *vs.* total-day).

**Table 1:** Characteristics of Participants by Activity Monitored, Mean (SD) or Percent.

Characteristics	Activity Monitored	
	In-School ^a^	Total-Day ^b^
N	191	150
Age (years)	4.1 (0.7)	4.1 (0.6)
BMI (kg/m^2^)	16.4 (2.3)	16.3 (1.9)
Gender (%)		
Boys	49.7	48.7
Girls	50.3	51.3
Race (%)		
African American	45.6	48.0
White	42.7	39.3
Other	11.5	12.7
Wear Time **^c^**		
Number of Days	4.6 (0.5)	6.6 (0.5)
Hours per Day	8.4 (1.4)	12.1 (2.6)

**^a^** Sedentary behavior monitored during school hours; **^b^** Sedentary behavior monitored during and after school hours; **^c^** Number of days and number of hours that children wore accelerometers.

### 3.2. Variability of Sedentary Behavior

The univariate analyses showed that the average time spent in sedentary behavior varied from 32 min/h to 51 min/h for both in-school and total-day depending upon the cutpoints used (Table 2). Regardless of cutpoints, CoV was greater for in-school sedentary behavior than total-day sedentary behavior, which indicates greater between-individual variability for in-school sedentary behavior compared to total-day sedentary behavior.

**Table 2:** Minimum, Maximum, Mean (SD), and Coefficient of Variance of Sedentary Behavior.

Cutpoints	Activity Monitored	Sedentary Behavior (min/h)			CoV (%)
		Min	Max	Mean (SD)	
**<37.5 cts/15 s**	In-school	13.4	46.9	32.1 (6.6)	20.6
	Total-day	19.6	40.0	32.3 (3.4)	10.7
**<200 cts/15 s**	In-school	21.2	55.8	44.5 (5.8)	13.1
	Total-day	33.7	51.1	45.6 (2.8)	6.1
**<373 cts/15 s**	In-school	42.1	58.1	51.2 (3.2)	6.3
	Total-day	41.7	55.0	51.3 (2.2)	4.2

CoV, Coefficient of Variance.

Gender and preschool-adjusted mean differences in sedentary behavior (min/h) across days of the week are presented in Table 3. There were no significant differences in min/h of in-school sedentary behavior across weekdays. For total-day sedentary behavior, however, post-hoc tests showed that preschool children spent less time in sedentary behavior on weekend days compared to weekdays (*P* < 0.05).

### 3.3. Reliability of In-School Sedentary Behavior

The ICCs for in-school sedentary behavior when applying three different accelerometry cutpoints are shown in Table 4. The ICCs were calculated for 1 day, 3 days, and 5 days. Across three different cutpoints, the ICCs ranged from 0.48 to 0.64, from 0.73 to 0.84, and from 0.82 to 0.90 for 1 day, 3 days, and 5 days, respectively. The number of days of accelerometry monitoring required to achieve the given levels of ICC (≥0.7, ≥0.75, ≥0.8, and ≥0.9) was calculated for in-school sedentary behavior (Table 4). The lowest number of days needed for the ICC of ≥0.8 was 2 days if used the cutpoint of <200 cts/15 s. Across three different cutpoints, between 2 and 4 days of monitoring was needed to achieve an ICC of ≥0.8.

**Table 3:** Accelerometry-derived Time Spent in Sedentary Behavior across Days of the Week, Mean (SE).

Day of the Week	Sedentary Behavior Cutpoints					
	<37.5 cts/15 s		<200 cts/15 s		<375 cts/15 s	
	Sedentary Behavior (min/h)		Sedentary Behavior (min/h)		Sedentary Behavior (min/h)	
	In-School	Total-Day	In-School	Total-Day	In-School	Total-Day
**MON**	32.9 (0.69)	33.2 (0.48)	44.7 (0.52)	46.0 (0.29)	51.4 (0.36)	51.6 (0.29)
**TUE**	33.3 (0.68)	33.1 (0.48)	45.3 (0.50)	46.2 (0.29)	51.6 (0.35)	51.7 (0.29)
**WED**	32.5 (0.68)	32.7 (0.47)	44.9 (0.51)	45.8 (0.28)	51.5 (0.35)	51.5 (0.29)
**THU**	33.7 (0.67)	33.5 (0.47)	45.6 (0.51)	46.6 (0.28)	51.9 (0.35)	52.0 (0.29)
**FRI**	32.0 (0.71)	32.8 (0.48)	44.3 (0.53)	45.9 (0.29)	51.1 (0.37)	51.5 (0.30)
**SAT**		30.4 (0.48)		44.1 (0.29)		50.5 (0.30)
**SUN**		31.4 (0.48)		45.0 (0.29)		51.0 (0.29)
**Average**	32.9	32.4	45.0	45.7	51.5	51.4
***P* for trend ^a^**	0.19	<0.05 ^b^	0.17	<0.05 ^b^	0.35	<0.05 ^b^

**^a^** Gender and preschool-adjusted repeated measures ANOVA; **^b^** Pairwise comparisons showed that SAT and SUN differ from MON–FRI, *P* < 0.05.

**Table 4:** ICCs, Number of Days to Achieve Desired ICCs, and SEMs According to Three Different Cutpoints.

In-School (*N* = 191)	ICCs ^a^			Number of Days for the Following ICCs ^c^				SEMs ^d^				
Cutpoints	1 d	3 d	5 d	0.7	0.75	0.8	0.9	1 d	2 d	3 d	4 d	5 d
<37.5 cts/15 s	0.51	0.76	0.84	2	3	4	9	4.6	3.7	3.2	2.9	2.6
<200 cts/15 s	0.64	0.84	0.90	1	2	2	5	3.5	2.7	2.3	2.0	1.8
<373 cts/15 s	0.48	0.73	0.82	2	3	4	10	2.3	1.9	1.7	1.5	1.4
**Total-Day (*N* = 150)**	**ICCs ^b^**			**Number of Days for the Following ICCs ^c^**				**SEMs ^d^**				
**Cutpoints**	**1 d**	**4 d**	**7 d**	**0.7**	**0.75**	**0.8**	**0.9**	**1 d**	**4 d**	**6 d**	**7 d**	**9 d**
<37.5 cts/15 s	0.32	0.65	0.76	5	6	9	19	2.8	2.0	1.8	1.7	1.5
<200 cts/15 s	0.36	0.69	0.80	4	5	7	16	2.2	1.6	1.3	1.3	1.2
<373 cts/15 s	0.38	0.71	0.81	4	5	6	14	1.7	1.2	1.0	1.0	0.9

**^a^** ICCs for the 1d, 3d, and 5d of in-school sedentary behavior; **^b^** ICCs for the 1 d, 4 d, and 7 d of total day sedentary behavior; **^c^** Number of days of monitoring required to achieve the ICCs of 0.7, 0.75, 0.8, and 0.9; **^d^** The unit of SEMs is min/h of sedentary behavior.

### 3.4. Reliability of Total-Day Sedentary Behavior

The number of days of accelerometry monitoring required to achieve the given levels of ICC (≥0.7, ≥0.75, ≥0.8, and ≥0.9) was calculated for total day sedentary behavior (Table 4). For total-day sedentary behavior, the ICCs were calculated for 1 day, 4 days, and 7 days. Across three different cutpoints, the ICCs ranged from 0.32 to 0.38, from 0.65 to 0.71, and from 0.76 to 0.81 for 1 day, 4 days, and 7 days, respectively. The lowest number of days needed for the ICC of ≥0.8 was 6 days if used the cutpoint of <373 cts/15 s. The number of days of accelerometry monitoring required to achieve an ICC of ≥0.8 was between 6 and 9 days across three different cutpoints.

### 3.5. SEMs for In-School and Total-Day Sedentary Behavior

The SEMs, the degree to which estimates of time spent in sedentary behavior (min/h) fluctuate as a result of measurement errors, are shown in Table 4. For in-school sedentary behavior, the SEMs were calculated for 1–5 days. Across three different cutpoints, the SEMs ranged from 2.3 to 4.6, from 1.7 to 3.2, and from 1.4 to 2.6 for 1 day, 3 days, and 5 days, respectively. For total-day sedentary behavior, the SEMs were calculated for 1–9 days. The SEMs ranged from 1.7 to 2.8, from 1.0 to 1.8, and from 0.9 to 1.5 for 1 day, 6 days, and 9 days, respectively.

## 4. Discussion

This is the first study to determine the number of days of accelerometry needed to reliably measure sedentary behavior in preschool children. We found that the reliability of accelerometry-derived sedentary behavior varied when three age-specific sedentary behavior cutpoints were applied. Our results suggest that, to achieve a reliability of 0.8, 2–4 days of monitoring are required to reliably measure in-school sedentary behavior, and 6–9 days of monitoring are required to reliably measure total day sedentary behavior in preschool children. The observed differences in the number of days needed to reliably measure sedentary behavior possibly attributed to different estimates of sedentary behavior across three different cutpoints. These findings have important implications for the measurement of sedentary behavior in preschool children using accelerometry.

A unique aspect of this study was that the reliability of accelerometry for measuring sedentary behavior was assessed separately for in-school and total day sedentary behavior. To our knowledge, only one previous study has determined the reliability of accelerometery for measuring children’s MVPA at specific times during the day (e.g., pre-, during-, and after school hours) [35], and no study has determined the reliability of accelerometry for measuring sedentary behavior. Estimating the specific reliability of in-school sedentary behavior is important because interventions designed to reduce sedentary behavior are often childcare center- or preschool-based [51,52,53,54,55], and researchers need a reliable measure to quantify their outcome of interest. Our results showed that in-school sedentary behavior was more stable than total-day sedentary behavior across days of the week, as evidenced by higher ICCs and the lower number of days required to achieve 80% reliability for in-school sedentary behavior compared to total day sedentary behavior.

We found that time spent in total-day sedentary behavior was significantly lower on weekend days compared to weekdays. This indicates that there was greater within-individual (day-to-day) variability in total-day sedentary behavior compared to in-school sedentary behavior [56,57]. By including the after-school hours and weekend data in total sedentary behavior, the within-individual variability of total-day sedentary behavior could be greater than that of in-school sedentary behavior. When we tested this hypothesis using our data, we observed the greater within-individual variability in total-day sedentary behavior compared to in-school sedentary behavior (data not shown). Therefore, the observed greater number of days of monitoring to achieve the desired reliability for total day sedentary behavior compared to in-school sedentary behavior is likely due to the greater within-individual variability in total day sedentary behavior.

It is also important to discuss our finding regarding the markedly longer monitoring period (14–19 days across different cutpoints) required to achieve ≥90% reliability for total day sedentary behavior. The formula used to estimate the ICCs is the function of between- and within-individual variability. As indicated by the low between-individual variability in our sample of preschool children, the ICCs for total-day sedentary behavior may be underestimated, which resulted in an overestimation of the number of days required to reliably measure sedentary behavior. Supporting this, the standard deviation and the coefficient of variance (CoV) were lower for total day sedentary behavior compared to in-school sedentary behavior (Table 2), suggesting the distribution of time spent in total sedentary behavior was more homogeneous than in-school sedentary behavior.

In addition to the reliability estimates observed in this study, we also presented the precision of the accelerometry-derived sedentary behavior using SEM. Unlike the observed reliabilities, the precision determined by SEMs was greater (*i.e.*, smaller SEM) for total-day compared to in-school sedentary behavior. This was due to the smaller standard deviation for total-day sedentary behavior as opposed to in-school sedentary behavior. Between different cutpoints, moreover, the precision determined by SEMs was not consistent with the reliability determined by ICCs. The disagreement between observed reliability (ICCs) and precision (SEMs) implies that understanding the reliability and precision of measurement of sedentary behavior is complex, and there is no single gold-standard statistical method to determine the number of days needed to reliably measure sedentary behavior. Nonetheless, both estimated ICCs and SEMs suggest that 2–4 days and 6–9 days of accelerometry monitoring provides a reliable and precise measure for in-school (ICCs ≥0.8; SEMs range: 1.5–3.7) and total-day sedentary behavior (ICCs ≥0.8; SEMs range: 0.9–1.8), respectively. 

These findings have important implications for both practitioners and researchers in public health. Based on our findings, two days of sedentary behavior monitoring may be acceptable for practitioners who use accelerometry to screen levels of preschooler’s sedentary behavior in childcare centers or preschools. A two-day monitoring reduces overall surveillance burden, such as time, costs, and technical expertise in comparison to five days of monitoring. From a research standpoint, however, selecting a protocol comparable with existing studies for comparative purposes and study resources needs to be considered in selecting the number of days of measurement. 

This study had several strengths, including the use of the most widely-validated accelerometers to measure sedentary behavior in preschool children [32,40,41,42]. Finding of this study are applicable to studies using different models of ActiGraph accelerometers because there is no inter-model differences in activity counts data [58,59]. This study also included the largest sample of preschool children among studies that have examined the reliability of accelerometry for the measurement of sedentary behavior in preschool children [36,60]._ENREF_25 The sample for this study, which consisted of subsamples of a voluntary sample of preschool children, may limit the generalizability of the findings of this study. Future research including different samples of preschool children should be explored to confirm the findings of this study.

## 5. Conclusions

In conclusion, depending upon the sedentary behavior cutpoints, two to four days and six to nine days of accelerometry monitoring are required to reliably measure in-school and total-day sedentary behavior, respectively, in preschool children. Overall, fewer days of accelerometry monitoring are required to reliably measure in-school sedentary behavior compared to total-day sedentary behavior. The greatest reliability, and hence the lowest number of days, to reliably measure accelerometry-derived sedentary behavior, was obtained using the cutpoints of <200 cts/15 s for in-school sedentary behavior (two days), and <373 cts/15 s for total-day sedentary behavior (six days), respectively. These findings provide important guidance for the design of research in which accelerometry will be used to measure sedentary behavior in preschool children. In addition, knowing the reliability of accelerometry in measuring sedentary behavior will ultimately contribute to research designed to monitor and reduce population levels of sedentary behavior.
